# Cracking the Code: Genotype–Phenotype Correlation Models in Sarcoglycanopathies

**DOI:** 10.1002/acn3.70361

**Published:** 2026-03-19

**Authors:** Leonela Luce, Goknur Selen Kocak, José Verdú‐Díaz, Jorge Alonso‐Pérez, Kristl G. Claeys, Tanya Stojkovic, Gorka Fernández‐Eulate, Pascal Laforêt, Najoua Miladi, Filipe Di Pace, Cristiane Araujo Martins Moreno, Edmar Zanoteli, Conrad C. Weihl, Volker Straub, Ana Töpf, Jordi Díaz‐Manera, Sarcoglycan European Cohort Consortium, Adele D′Amico, Adolfo López de Munain, Alicia Alonso‐Jiménez, Ana Camacho‐Salas, Andrea Gangfuß, Andrés Nascimento, Anna Sarkozy, Anneke J. van der Kooi, Arturo Fraga‐Bau, Béla Melegh, Benedikt Schoser, Bjarne Udd, Blaz Koritnik, Carlos Ortez, Chiara Marini Bettolo, Chiara Panicucci, Claudia Weiss, Claudio Bruno, Claudio Semplicini, Cristina Dominguez‐González, Cristina Garrido, David Gómez‐Andrés, Edoardo Malfatti, Elena Pegoraro, Elke De Vos, Francina Munell, Gabriele Dekomien, Giacomo Pietro Comi, Giorgio Tasca, Isabelle Richard, Jan L. De Bleecker, Jana Haberlová, Jesper Helbo Storgaard, Johanna Palmio, John Vissing, Juan Carlos de Leon‐Hernández, Kinga Hadzsiev, Laura Costa‐Comellas, Lea Leonardis, Leroy ten Dam, Lidia González‐Quereda, Luca Bello, Luisa Politano, Manuela Santos, Marianne de Visser, Marie Rohlenová, Matteo Garibaldi, Michela Guglieri, Nicolas Deconinck, Nicoline Løkken, Omar Abdel‐Mannan, Pia Gallano, Roberto Fernández‐Torrón, Ulrike Schara‐Schmidt, Vincenzo Nigro, Vittoria Zangaro

**Affiliations:** ^1^ John Walton Muscular Dystrophy Research Centre, Translational and Clinical Research Institute Newcastle University, and Newcastle Hospitals NHS Foundation Trust Newcastle upon Tyne UK; ^2^ Neuromuscular Disease Unit, Neurology Department Hospital Universitario Nuestra Señora De Candelaria. Instituto De Investigación Sanitaria De Canarias (IISC) Tenerife Spain; ^3^ Department of Neurology University Hospitals Leuven Leuven Belgium; ^4^ Laboratory for Muscle Diseases and Neuropathies, Department of Neurosciences KU Leuven Leuven Belgium; ^5^ Centre De Référence Des Maladies Neuromusculaires Nord/Est/Île‐De‐France, APHP, Groupe Hospitalier Pitié‐Salpêtrière Sorbonne Université Paris France; ^6^ Centre De Référence Des Maladies Neuromusculaires Nord/Est/Île‐De‐France. APHP, CHU Raymond Poincaré, Garches Université Paris‐Saclay Paris France; ^7^ Faculty of Medicine of Tunis University of Tunis El Manarrue Du Docteur Hassouna Ben Ayed Tunis Tunisia; ^8^ Laboratoire De Recherche En Neurologie pédiatrique (LR18SP04) à L'institut National Mongi Ben Hamida De Neurologie Tunis Tunisia; ^9^ Maghreb Medical Center Tunis Tunisia; ^10^ Department of Neurology Faculdade De Medicina Da Universidade De São Paulo (FMUSP) São Paulo Brazil; ^11^ Department of Neurology Washington University School of Medicine Saint Louis Missouri USA

**Keywords:** disease progression, genotype–phenotype correlation, prognosis, sarcoglycanopathies

## Abstract

**Objective:**

Sarcoglycanopathies are among the most severe limb‐girdle muscular dystrophies (LGMD), though milder presentations have been described. These diseases are primarily caused by missense variants, but the limited predictability of their effect on protein maturation, complex formation, and transport has hindered reliable genotype–phenotype correlations. This study aimed to establish accurate genotype–phenotype correlations for LGMDR3, LGMDR4, and LGMDR5.

**Methods:**

We analyzed the largest sarcoglycanopathy cohort to date (*n* = 541). Clinical data, including age at symptom onset and loss of ambulation, were collected and used to define phenotype severity. Predictive models were developed considering the impact of non‐truncating variants on secondary structure, functional domains, evolutionary conservation, and intra‐complex protein–protein interactions. Patients carrying two variants predicted to disrupt membrane trafficking were expected to present with severe phenotypes.

**Results:**

For LGMDR3, the best‐performing model classified variants affecting β‐sheets, cadherin‐like, and ATP‐binding domains as disruptive to membrane translocation, achieving 89% predictive power, 0.867 balanced accuracy, and 2.4% false‐negative rate (clinically severe patients who were wrongly classified by the model as mild). For LGMDR4 and LGMDR5, the best‐performing model involved conserved residues, β‐sheets, EGF‐like domain, and protein–protein interactions—reaching 80% predictive power, 0.689 balanced accuracy, and 3.1% false‐negative rate (LGMDR4), and 90% predictive power, 0.536 balanced accuracy, with no false negatives (LGMDR5). Additionally, we developed an open‐access predictive tool for clinical and research application.

**Interpretation:**

This study provides a robust genotype–phenotype correlation for sarcoglycanopathies, improving prognosis, patient management, and clinical trial recruitment.

## 1 Introduction

Sarcoglycanopathies are among the most frequent and severe forms of limb‐girdle muscular dystrophy (LGMD) and, as such, they are characterized by progressive proximal muscle weakness of the scapular and pelvic girdles. The term comprehends four autosomal recessive disorders caused by pathogenic variants in the sarcoglycan genes: LGMDR3 (OMIM: 608099, *SGCA*, α‐sarcoglycan), LGMDR4 (OMIM: 604286, *SGCB*, β‐sarcoglycan), LGMDR5 (OMIM: 253700, *SGCG*, γ‐sarcoglycan), and LGMDR6 (OMIM: 601287, *SGCD*, δ‐sarcoglycan).

These conditions typically present with onset of symptoms in the first decade of life and loss of ambulation during adolescence or early adulthood. However, they exhibit a broad spectrum of severity and progression, and some milder cases can present with adult‐onset symptoms, exercise intolerance and/or rhabdomyolysis [1, 2, 3]. These milder forms are rare and more common in certain sarcoglycanopathy subtypes than others.

The sarcoglycan genes encode single‐pass transmembrane glycoproteins that are co‐translationally translocated into the endoplasmic reticulum (ER), where they get properly folded and matured. In this organelle the sarcoglycan (SC) complex is sequentially assembled: (i) β‐sarcoglycan strongly interacts with δ‐sarcoglycan, giving rise to the βδ‐core; (ii) γ‐sarcoglycan associates with the βδ‐core; and (iii) α‐sarcoglycan forms weak bonds with γ‐sarcoglycan [4, 5]. The resulting heterotetramer is a component of the dystrophin‐associated glycoprotein complex (DAG), which is located in the membrane of skeletal and cardiac muscle fibers. It connects the intracellular cytoskeleton to the extracellular matrix, protecting the fiber from contraction‐induced damage. Additionally, α‐sarcoglycan has been reported to present ecto‐ATPase activity, suggesting a role in signaling and modulation of cellular pathways [6, 7].

Pathogenic variants in any of the sarcoglycan genes tend to disrupt the assembly and transport of the entire SC complex, with its absence or reduction at the membrane being the principal underlying cause of LGMD. Although immunohistochemistry (IHC) and western blot (WB) tests performed on muscle tissue samples are key elements to confirm the clinical suspicion of Sarcoglycanopathies, studies have shown little to no correlation between staining patterns and the specific gene affected, with some cases even displaying abnormal results for all four proteins [8, 9, 10]. Moreover, normal IHC results do not rule out the presumptive clinical diagnosis and, therefore, cannot attest to the pathogenicity of the identified variants. Among all the different variants reported in the Leiden Open Variation Database (LOVD) for these genes, 43%–57% are missense changes, which are thought to result in the production of a functionally altered protein with preserved membrane localization [11]. Although this has been confirmed for some missense variants, others have been shown to disrupt membrane localization [12]. Recently, a study using high‐throughput mutagenesis of all β‐sarcoglycan residues demonstrated that variants affecting β‐sheets are more likely to impair membrane localization and, therefore, pathogenic [13].

The limited predictability of how variants affect protein maturation, complex formation, and transport has hindered robust genotype–phenotype correlations. Such correlations are essential for identifying patients who require more frequent follow‐up, improving the information provided to patients in terms of disease prognosis expectations, helping to deliver high‐standard, individualized, specialized care, and optimizing patient selection for clinical trials.

Given the clinical relevance of establishing accurate genotype–phenotype correlations, our aim was to identify optimal models for predicting disease severity in LGMDR3, LGMDR4, and LGMDR5. To achieve this, we assembled one of the largest sarcoglycanopathy cohorts to date and compared patients' disease progression with phenotype predictions generated by various models considering the impact of non‐truncating variants on secondary structure, functional domains, evolutionary conservation, and intra‐complex protein–protein interactions.

## 2 Materials and Methods

### 2.1 Study

This retrospective observational study collected de‐identified, pre‐existing clinical and genetic data from patients with confirmed genetic diagnosis of LGMDR3, LGMDR4 or LGMDR5. We excluded LGMDR6 due to its extreme rarity and predominant association with truncating variants. De‐identified patient data were collected from 37 centres across 19 countries. While most information came from European centres and was previously compiled for Alonso‐Pérez et al., 2020 [14] and for Guimerães‐Costa et al., (2020) [9], we also included data from American (Brazil and the United States) and African (Tunisia) centres. Informed consent had been obtained from all participants at local sites under the relevant ethical approvals.

### 2.2 Patients and Data Collection

Participating centres completed a survey detailing demographics, clinical history, WB, and genetic findings, as described in Alonso‐Pérez et al., 2020 [14]. Inclusion criteria were as follows: (i) individuals carrying homozygous or assumed compound heterozygous pathogenic, likely pathogenic, or variants of unknown significance in *SGCA, SGCB*, or *SGCG*, curated according to the LGMD variant curation expert panel specifications; and (ii) individuals with sufficient phenotypic data to determine time to loss of ambulation and/or disease duration. Exclusion criteria were as follows: (i) individuals carrying a single heterozygous variant in any sarcoglycan gene; (ii) individuals carrying homozygous or assumed compound heterozygous for benign or likely benign variants or for splice site variants in the above‐mentioned genes; and/or (iii) individuals with incomplete clinical data. We decided to exclude both canonical and non‐canonical splice site variants because their effects on transcript maturation—and consequently on protein sequence and structure—are difficult to predict accurately using computational tools alone, and this uncertainty could negatively impact model performance.

### 2.3 Clinical Analysis

We established the following definitions for the clinical analysis: (1) Age at onset, age at which the first signs or symptoms were noticed by the patients or their parents, regardless of their type; (2) Age at loss of ambulation, age at which patients were no longer able to walk short distances (< 10 m) and required continuous wheelchair use; (3) Time to loss of ambulation, period from symptom onset to loss of ambulation; and (4) Disease duration, period from symptom onset to the last clinical evaluation [14].

We then classified patients according to time to loss of ambulation into three groups: Severe, Typical, and Mild. Classification boundaries were determined using Kaplan–Meier analysis, which showed that 50% of patients lost ambulation 14.0 ± 0.3 years after onset, with the 25th percentile at 11 years and the 75th percentile at 19 years. Accordingly, phenotypes were considered severe if patients lost ambulation within 11 years (< 11 y) from onset, typical if ambulation was lost between 11 and 19 years (≥ 11 y and < 19 y) from onset, and mild if patients remained ambulant for more than 19 years (≥ 19y) from the age at onset. Moreover, patients with non‐pediatric onset (older than 16 years of age) and/or those who remained ambulant at ages older than 35 years were classified as mild, regardless of disease duration. Ambulant patients with disease onset before 16 years of age and a disease duration of less than 19 years could not be classified and were therefore excluded from the analysis.

### 2.4 Sarcoglycan Complex Structure Modeling and Schematic Summaries

**FIGURE 1:**
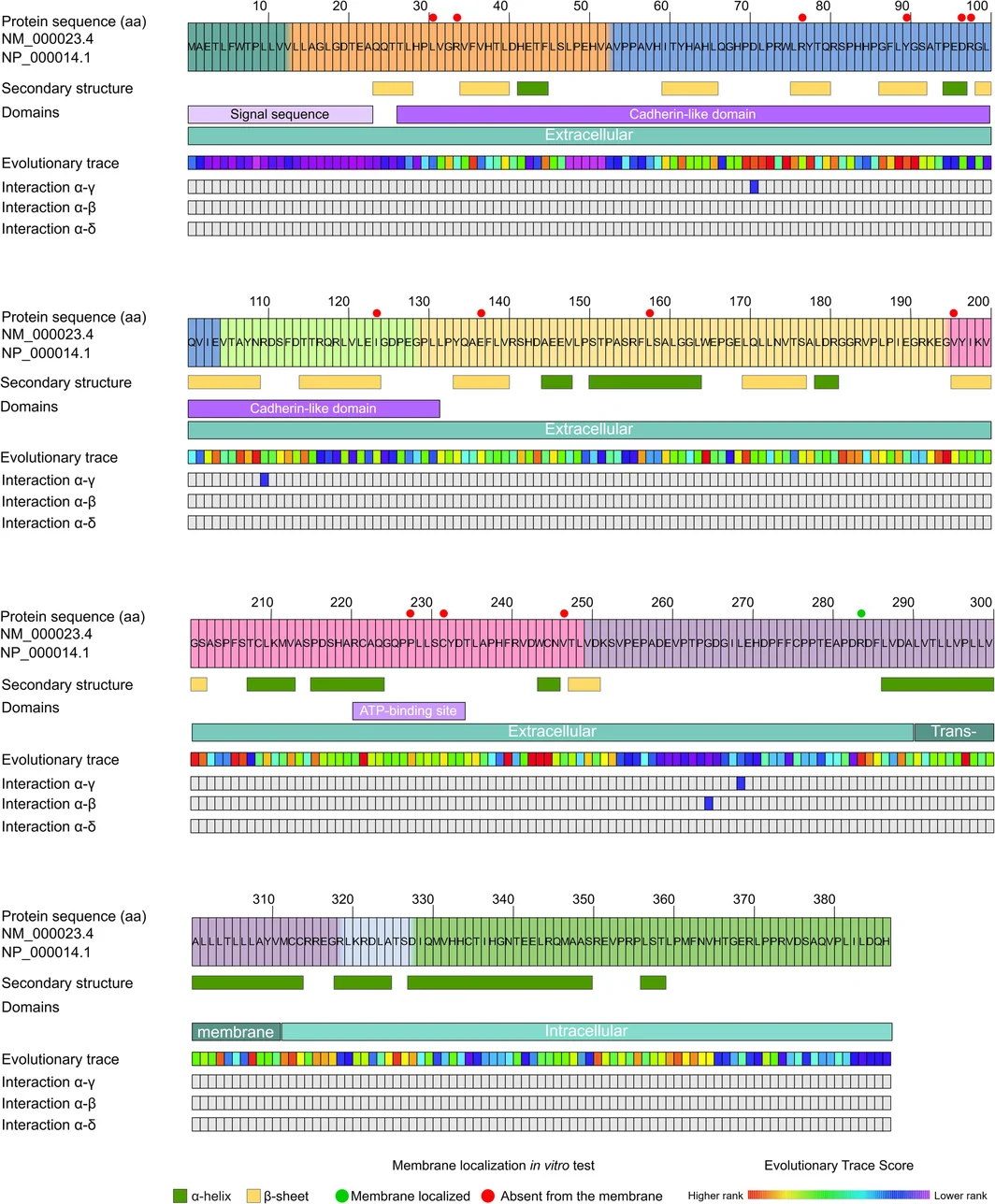
α‐sarcoglycan schematic summary. Representation of the α‐sarcoglycan protein sequence, with each residue depicted as a rectangle labeled with its one‐letter amino acid code. The figure integrates the following structural and functional information: (I) residues functionally tested *i* *n* *v* *itro*—those impairing membrane translocation (red circles above the amino acid sequence) and those permitting it (green circles above); (ii) secondary structure elements—α‐helices (green) and β‐sheets (yellow); (iii) functional domains (various shades of purple); (vi) structural domains (various shades of green); (v) Evolutionary Trace ranking—highly conserved residues in red and poorly conserved in purple; (vi) residues involved in intra‐complex protein–protein interactions (blue). The position and length of each colored block indicate the specific amino acids involved in each structural or functional feature.

Secondary structures of α‐, β‐, and γ‐sarcoglycan within the complex were predicted using the multimer algorithm from AlphaFold3 and the protein sequences of the four sarcoglycans available in Uniprot (Q16586, Q16585, Q13326, and Q92629) [15]. Five models were obtained as output and were rendered with ChimeraX (Figure S1) [16, 17]. Since the confidence metrics were similar across models, we created schematic summaries for each gene, defining the secondary structures and protein–protein interactions as those shared by most AlphaFold models (Figures 1, S2 and S3). Additionally, the schematic summaries included information regarding: (i) structural domains (intracellular, transmembrane, and extracellular); (ii) functional domains (ATP‐binding, cadherin‐like, and EGF‐like domains, and signal sequence); (iii) residues where missense variants have been previously functionally tested in vitro to determine their impact on protein translocation to the membrane; and (iv) a ranking of the functional and structural importance of each residue among orthologs, calculated using the Evolutionary Trace method [18].

### 2.5 Analysis of Genetic Data

Variants were described according to HGVS guidelines and using the MANE SELECT transcripts (NM_000023 (*SGCA*), NM_000232 (*SGCB*), and NM_000231 (*SGCG*)). Variants were classified by type and effect into the following categories: start or stop loss, missense, nonsense, small in‐frame or out‐of‐frame deletions/duplications/insertions/delins, and in‐frame or out‐of‐frame exonic deletions/duplications (1 or more full exons). Variants were further grouped into truncating (nonsense, start loss, and out‐of‐frame variants) and non‐truncating (missense and in‐frame variants).

We implemented multiple databases, bioinformatic tools, literature reviews and the schematic summaries to compile the following information: ClinVar classification, affected secondary structure, residue conservation across species, intra‐complex protein–protein interactions, IHC results from homozygous individuals and functional assay data (Table S1).

### 2.6 Genotype–Phenotype Predictions

Variants were classified as: (1) Predicted to be absent from the membrane (AM), variants impairing complex formation and membrane localization; and (2) Predicted to be localized at the membrane (LM), variants preserving protein transport to the membrane. We developed and evaluated the performance of multiple models generated by combining four different terms: (1) Secondary structure (β‐sheet vs. β‐sheet+α‐helix); (2) Functional domains (Cadherin‐like, ATP‐binding, or EGF‐like domains, depending on the gene); (3) Residue conservation; and (4) Intra‐complex protein–protein interactions. The classification and the predicted outcome varied depending on the model being tested; thus, the same variant could be classified as AM or LM depending on the model evaluated. For example, missense variants affecting *SGCA* residues 66 and 67 were classified as AM and LM, respectively, by a model considering only β‐sheets, but were both classified as AM when the functional domain term was included in the model (Figure 1). Phenotype predictions were then derived from genotype classifications: individuals with AM/AM genotypes were expected to have Severe or Typical presentations, while those with AM/LM or LM/LM genotypes were expected to present Mild phenotypes.

Model's performance was evaluated using the following parameters: (1) Predictive power (PP), percentage of individuals for whom the expected and observed phenotype matched; (2) False‐positive (FP) rate, percentage of individuals predicted to have a severe or typical phenotype (AM‐AM), but who exhibited a mild phenotype; (3) False‐negative (FN) rate, percentage of individuals predicted to be mild (AM‐LM or LM‐LM), but who exhibited a severe or typical phenotype; and (4) Balanced accuracy (BA), average of sensitivity and specificity, a parameter adjusted for class imbalance—required here due to the presence of recurrent and highly frequent variants in the sarcoglycan genes. The best‐performing model was chosen based on the highest BA. However, when multiple models had similar scores, the one with the lowest FN rate was selected.

## 3 Results

### 3.1 Study Cohort and Clinical Analysis

We collected clinical and genetic data from 541 patients, including 221 LGMDR3, 88 LGMDR4, and 232 LGMDR5. After excluding cases that could not be accurately classified clinically and/or genetically, we conducted a genotype–phenotype correlation analysis with a refined cohort of 316 patients (124 LGMDR3, 65 LGMDR4, and 127 LGMDR5).

Patients were phenotypically classified as severe, typical, or mild according to the time to loss of ambulation, as described above. In the LGMDR3 group, 51 patients were classified as severe, 14 as typical, and 59 as mild. In the LGMDR4 group, 32 patients were categorized as severe, 14 as typical, and 19 as mild. Lastly, for LGMDR5 patients, 83 were categorized as severe, 30 as typical, and 14 as mild.

### 3.2 Phenotype Distribution According to Variant Type

**FIGURE 2:**
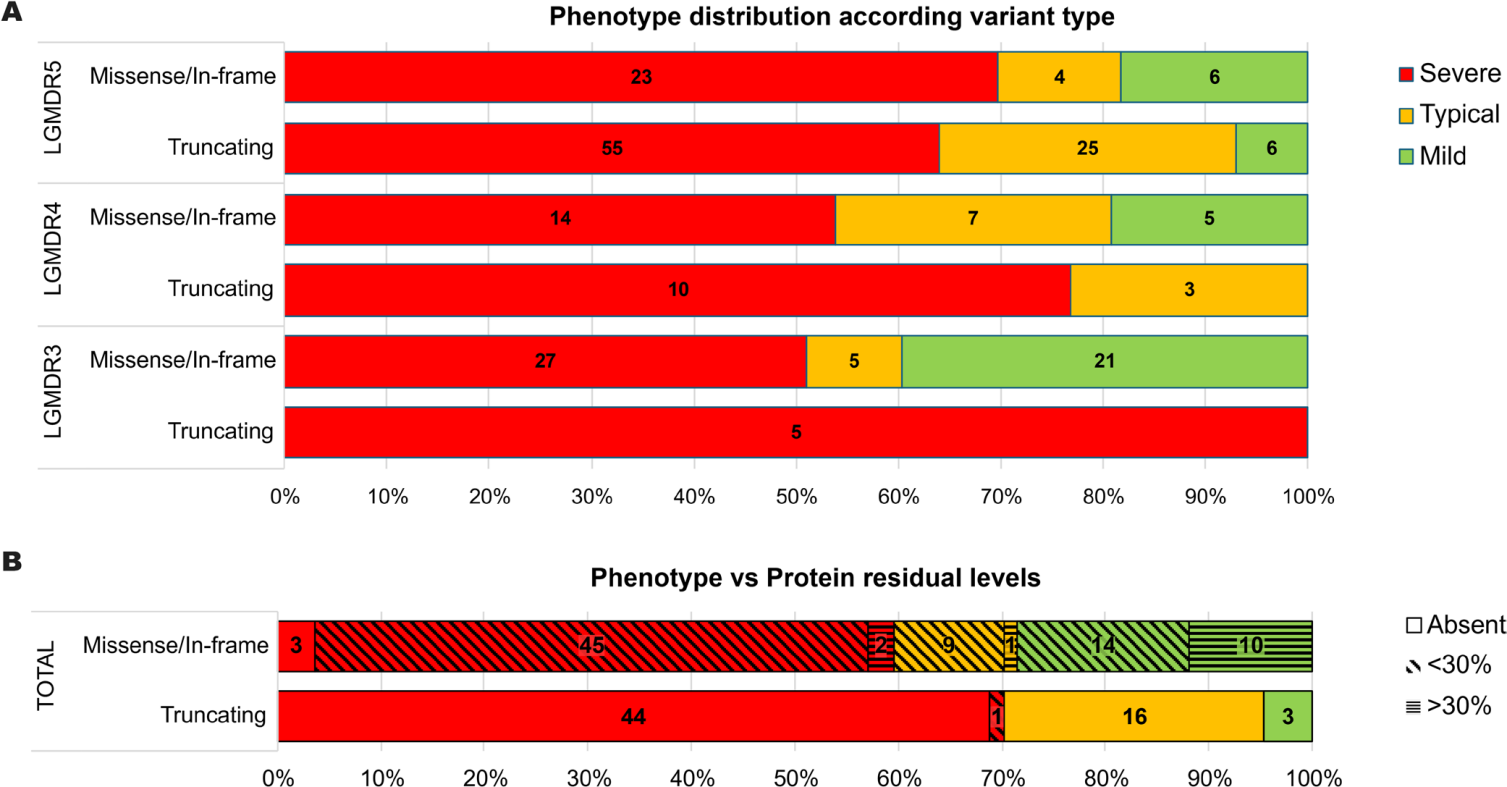
Phenotype distribution according to variant type and residual protein levels. Stacked bar charts showing: (A) phenotype distribution among homozygous patients carrying truncating and non‐truncating (missense or in‐frame) variants for LGMDR3, LGMDR4, and LGMDR5; (B) residual protein levels on western blot and phenotype distribution among homozygous patients with truncating or non‐truncating variants in the analyzed sarcoglycan genes. Numbers within each bar segment represent the number of cases per phenotype category (Severe—red; Typical—orange; Mild—green) or residual protein level (Absent—filed; < 30% oblique black lines; > 30% thin horizontal black lines).

Firstly, we tested the hypothesis that non‐truncating variants, such as missense or in‐frame variants, potentially lead to milder phenotypes as they theoretically produce proteins with reduced functionality but preserved membrane localization. In contrast, truncating variants are expected to result in severe or typical phenotypes, as they likely lead to complete loss of expression and protein function [14, 19]. To evaluate this, we analyzed the phenotypes of homozygous patients carrying either truncating or non‐truncating variants. Truncating variants predominantly led to severe or typical phenotypes (100% for LGMDR3 and LGMDR4, and 93.0% for LGMDR5) (Figure 2A). Non‐truncating variants exhibited a broader phenotypic spectrum, very often resulting in unexpected phenotypes (60.4% for LGMDR3, 80.8% for LGMDR4, and 81.8% for LGMDR5), highlighting the need for refined predictive models capable of distinguishing between variants that cause mild or severe disease phenotypes.

Additionally, we examined the relationship between phenotype and residual sarcoglycan protein levels on WB. Regardless of the patients' phenotype, 98.4% of cases with truncating variants were associated with absent sarcoglycan expression (Figure 2B). In contrast, non‐truncating variants principally resulted in a severe reduction of sarcoglycan levels (< 30%), independent of the patient's clinical presentation. Similar results were observed for LGMDR3, LGMDR4, and LGMDR5 (data not shown). All cases with > 30% residual protein expression were associated with non‐truncating variants, and 76.9% were observed among mild patients.

### 3.3 Models Based on Secondary Structure

**FIGURE 3:**
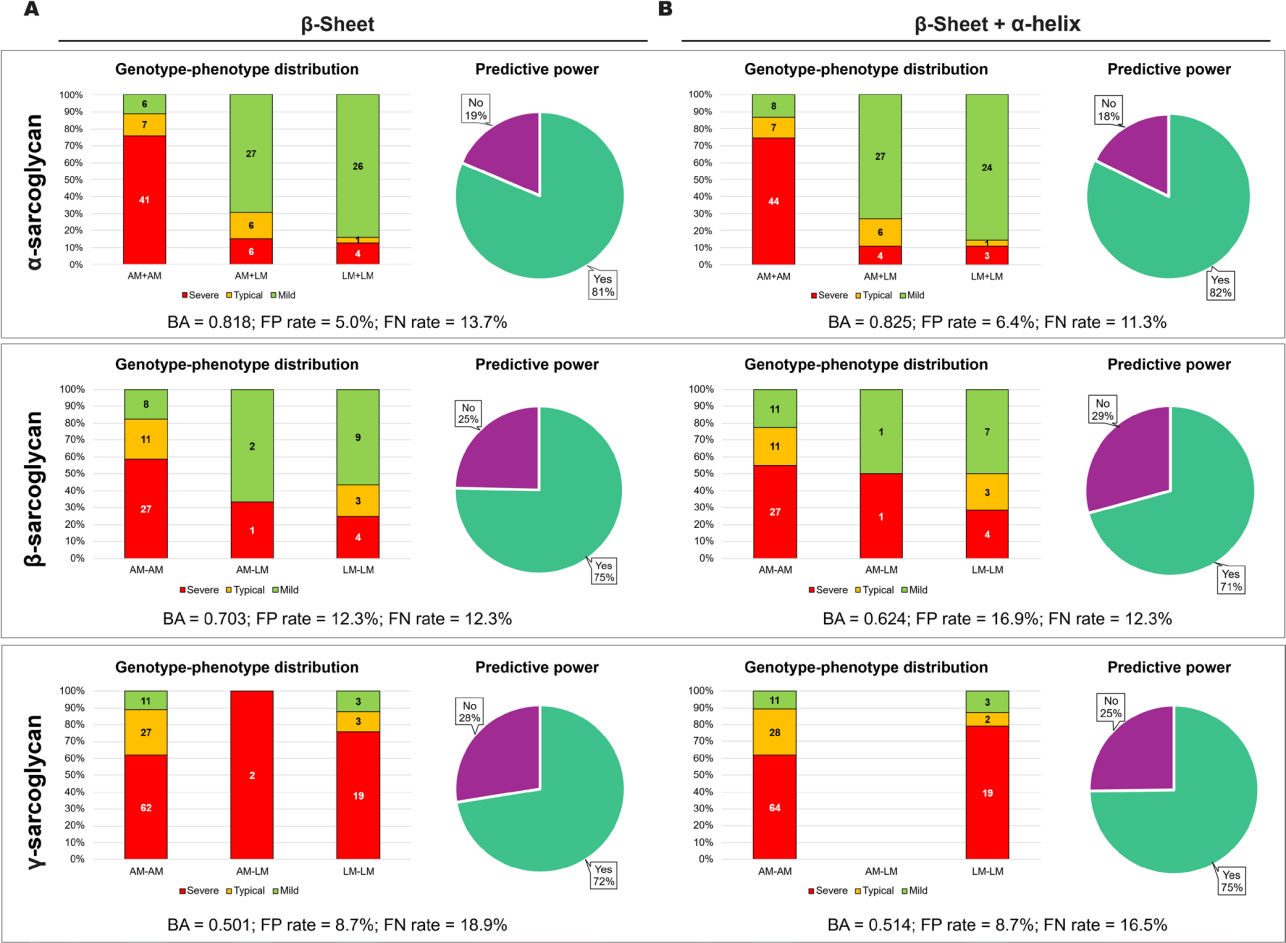
Genotype–phenotype correlation models based on secondary structure. Performance evaluation of genotype–phenotype correlation models incorporating: (A) β‐sheet only and (B) β‐sheet plus α‐helix structural features, across each analyzed sarcoglycan gene. Bar plots show the distribution of observed clinical phenotypes—Severe (red), Typical (orange), and Mild (green)—within each predicted genotype group: AM‐AM (two variants predicted to impair membrane localization), AM‐LM (one impairing and one preserving membrane localization), and LM‐LM (two variants preserving membrane localization). Numbers within each bar segment represent the number of cases per phenotype category. Corresponding pie charts summarize the overall predictive power of each model, indicating the proportion of cases in which the predicted genotype agrees with the observed clinical phenotype. AM: Absent from the membrane; BA: Balanced accuracy; FP rate: False‐positive rate; FN rate: False‐negative rate; and LM: Localized at the membrane.

We initially developed models that classified variants affecting secondary structure elements (α‐helix and β‐sheet) as disruptive to the SC complex's ability to translocate to the membrane (AM). The simplest model considered only variants affecting β‐sheets, as the AlphaFold complex outputs suggested that these structures constitute the binding scaffolds of the triple‐helical quaternary structure shared by β‐, γ‐, and δ‐sarcoglycans (Figure S1). This model achieved PP and BA values of 81% and 0.818 for LGMDR3, 75% and 0.703 for LGMDR4, and 72% and 0.501 for LGMDR5 (Figure 3A). The phenotype distribution across AM‐AM, AM‐LM, and LM‐LM genotypes supports our prediction that the presence of one allele preserving sarcoglycan localization at the membrane is sufficient to result in a milder disease course. These results were used as a baseline for evaluating subsequent models.

Due to the limited number of variants affecting α‐helices, we did not test a model exclusively focused on them. Instead, we analyzed a combined model that classified variants in α‐helices or β‐sheets as AM. For LGMDR3 and LGMDR5, this model showed a slight increase in PP and BA compared to the β‐sheet‐only model (82% and 75%, 0.825 and 0.514, respectively; Figure 3B). This improvement was associated with a small decrease in the FN rates, from 13.7% to 11.3% for LGMDR3, and from 18.9% to 16.5% for LGMDR5. However, for LGMDR3, this combined model also led to an increase in the FP rate. In contrast, for LGMDR4, the combined model showed a lower PP (71%) and BA (0.624) compared to the β‐sheets‐only model.

### 3.4 Models Based on Functional Domains

We also tested models that combined the effects of variants on secondary structure and functional domains. Different models were constructed for α‐sarcoglycan and for β‐ and γ‐sarcoglycans as they are distinct types of membrane proteins with different domains.

**FIGURE 4:**
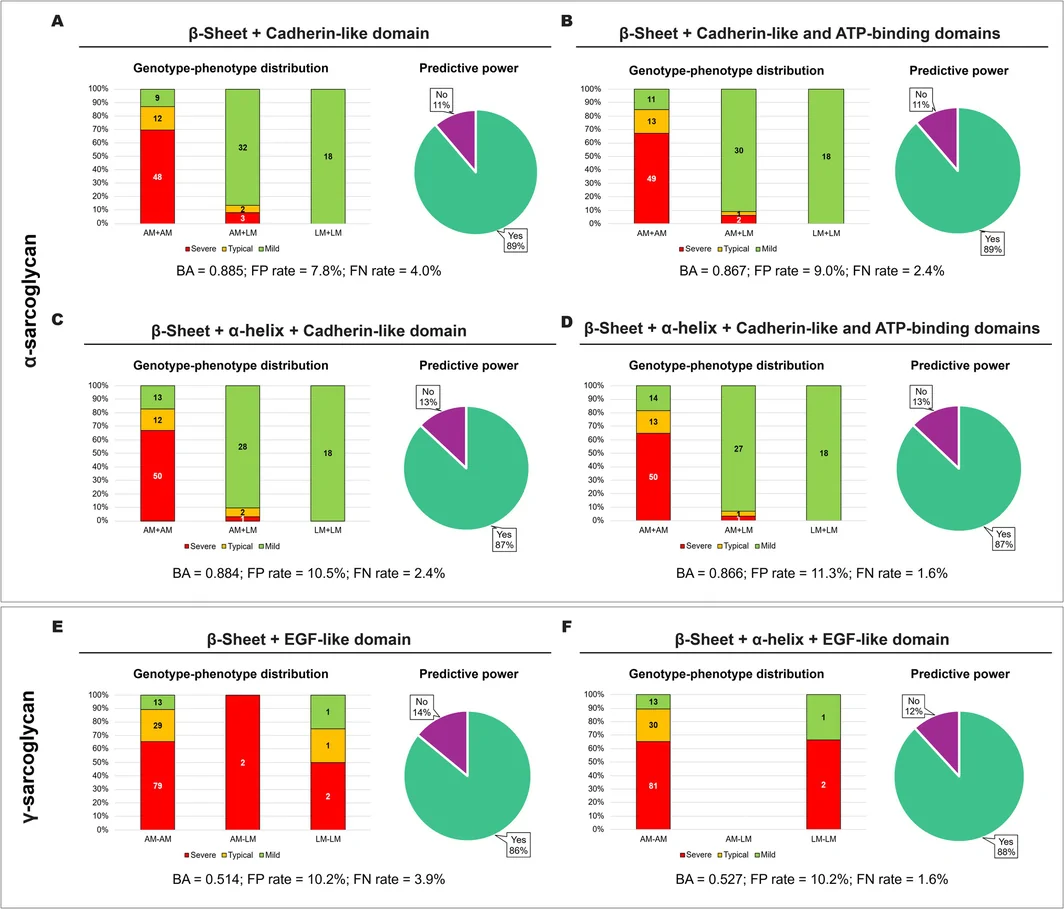
Genotype–phenotype correlation models integrating secondary structure and functional domains. Performance evaluation of genotype–phenotype correlation models incorporating combinations of secondary structure elements and functional domains. For α‐sarcoglycan, models include (A) β‐sheet and cadherin‐like domain, (B) β‐sheet plus cadherin‐like and ATP‐binding domains, (C) β‐sheet, α‐helix and cadherin‐like domain and (D) β‐sheet and α‐helix plus cadherin‐like and ATP‐binding domains. For γ‐sarcoglycan, models included: (E) β‐sheet and EGF‐like domain, and (F) β‐sheet, α‐helix and EGF‐like domain. Bar plots show the distribution of observed clinical phenotypes—Severe (red), Typical (orange), and Mild (green)—within each predicted genotype group (AM‐AM, AM‐LM and LM‐LM). Numbers within each bar segment represent the number of cases per phenotype category. Pie charts summarize the predictive power of each model, indicating the proportion of cases with concordant genotype and phenotype classifications. AM: Absent from the membrane; BA: Balanced accuracy; FP rate: False‐positive rate; FN rate: False‐negative rate; and LM: Localized at the membrane.

For LGMDR3, we investigated four models. The first one classified variants affecting β‐sheets and/or the cadherin‐like domain as AM. This model showed a major improvement in PP (89%) and BA (0.885) compared to the β‐sheet‐only model, primarily due to a marked reduction in the FN rate (4.0% and 13.7%, respectively) (Figure 4A). Yet, this model also increased the FP rate to 7.8%. The second model expanded on the first one by also considering changes affecting the ATP‐binding domain as AM variants. Although the PP remained the same as the previous model, the BA decreased to 0.867, as the further reduction in the FN rate (2.4%) was offset by an increase in the FP rate to 9.0% (Figure 4B). The third and fourth models were extensions of the first and second, respectively, incorporating variants affecting α‐helices in the AM definition. While both showed improved PP and BA relative to the baseline model, they did not outperform the β‐sheet+cadherin‐like domain nor the β‐sheet+cadherin‐like and ATP‐binding domains models, respectively (Figure 4C,D).

For LGMDR4, we were unable to evaluate the impact of variants in the EGF‐like domain alone, as all variants in this functional domain also mapped to β‐sheets. Therefore, the performance of this model mirrored that of the previous section (Figure 3). In contrast, this analysis was feasible for LGMDR5. The model that classified variants affecting β‐sheets and/or the EGF‐like domain as AM exhibited an improved PP (86%) and BA (0.514) compared to the β‐sheet‐only model (Figure 4E). However, it was outperformed by a model that also considered variants affecting α‐helices as AM, achieving a PP of 88% and a BA of 0.527 (Figure 4F). Though both models increased the FP rate (10.2%) and reduced the FN rate, the latter demonstrated a superior performance (1.6% vs. 3.9%, respectively).

### 3.5 Models Based on Conservation and Intra‐Complex Protein–Protein Interactions

**FIGURE 5:**
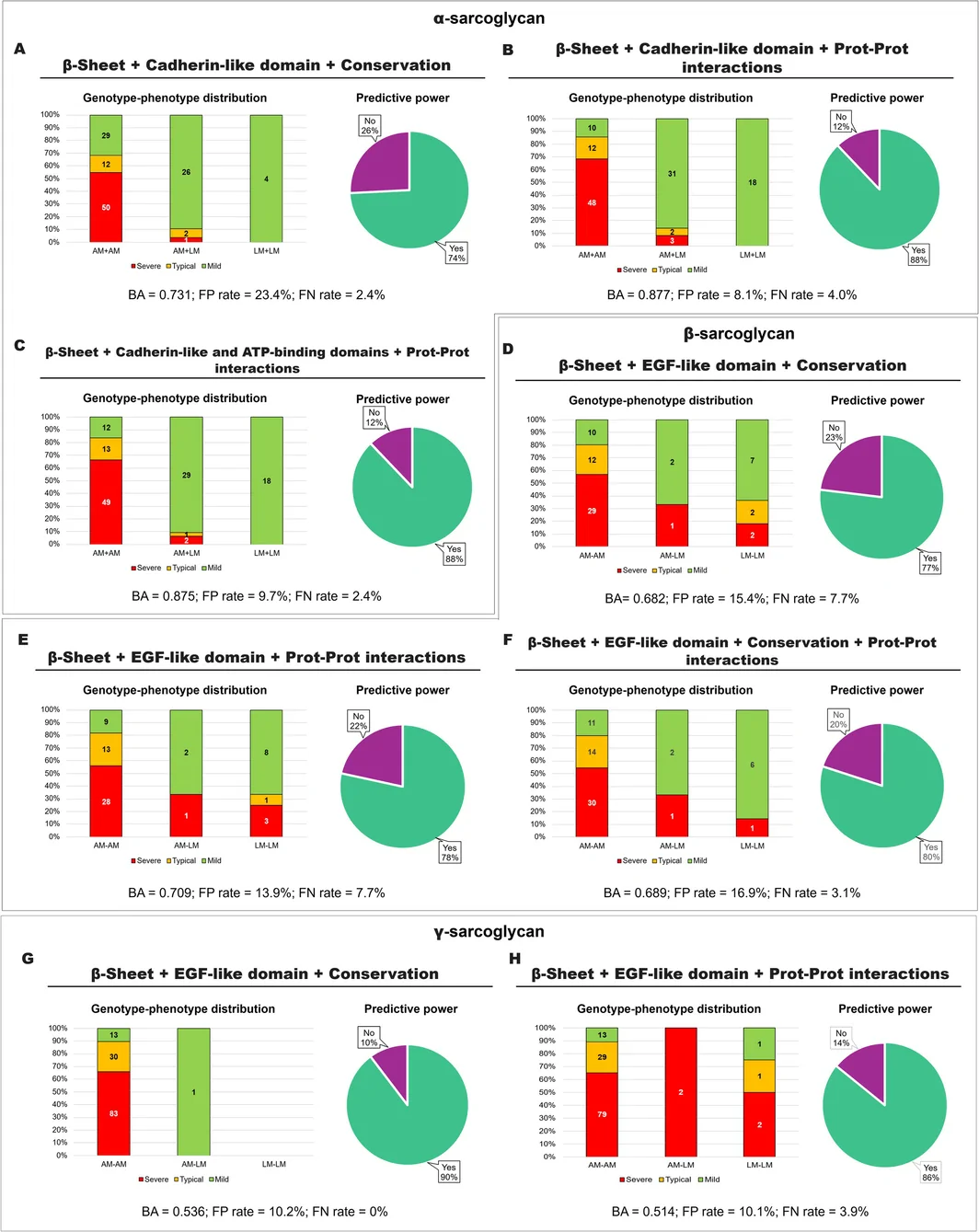
Genotype–phenotype correlation models integrating secondary structure, functional domains, conservation and protein–protein interactions. Performance evaluation of genotype–phenotype correlation models incorporating combinations of secondary structure elements, functional domains, conservation and protein–protein interactions. For α‐sarcoglycan, models include (A) β‐sheet, cadherin‐like domain and conservation, (B) β‐sheet, cadherin‐like domain and protein–protein interactions, and (C) β‐sheet, cadherin‐like and ATP‐binding domains plus protein–protein interactions. For β‐sarcoglycan, models include (D) β‐sheet, EGF‐like domain and conservation, (E) β‐sheet, EGF‐like domain and protein–protein interactions, and (F) β‐sheet, EGF‐like domain, conservation and protein–protein interactions. For γ‐sarcoglycan, models included: (G) β‐sheet, EGF‐like domain and conservation, and (H) β‐sheet, EGF‐like domain and protein–protein interactions. Bar plots show the distribution of observed clinical phenotypes—Severe (red), Typical (orange), and Mild (green)—within each predicted genotype group (AM‐AM, AM‐LM and LM‐LM). Numbers within each bar segment represent the number of cases per phenotype category. Corresponding pie charts summarize the predictive power of each model, indicating the proportion of cases in which the predicted genotype agrees with the observed clinical phenotype. AM: Absent from the membrane; BA: Balanced accuracy; FP rate: False‐positive rate; FN rate: False‐negative rate; and LM: Localized at the membrane.

For LGMDR3, we studied three models. The first classified variants affecting either β‐sheets, the cadherin‐like domain, and highly conserved residues, as well as any combination of the three, as AM. This model underperformed compared to the β‐sheet‐only baseline by increasing the FP rate to 23.4% (Figure 5A). We then tested two additional models incorporating variants affecting intra‐complex protein–protein interactions: one alongside β‐sheet and/or cadherin‐like domain variants, and the other including variants affecting either the β‐sheets, cadherin‐like and/or ATP‐binding domains. Both models slightly underperformed compared to their counterparts lacking the interaction term, each achieving a PP of 88% and BA scores of 0.877 and 0.875, respectively (Figure 5B,C).

For LGMDR4 and LGMDR5, we investigated two models built upon the β‐sheet and/or EGF‐like domain model by adding either a conservation or an intra‐complex protein–protein interaction term. In both diseases, the model with the conservation term outperformed the β‐sheet‐only baseline in terms of PP and FN rate, achieving 77% and 7.7% for LGMDR4, and 90% and 0% for LGMDR5, respectively (Figure 5D,G). The second model, which included the interaction term, underperformed the first one for γ‐sarcoglycan (Figure 5E,H). For LGMDR4, we also evaluated a combined model classifying variants affecting either β‐sheets, EGF‐like domain, conservation, and intra‐complex protein–protein interactions, as well as any combination of the four terms, as AM. While this combined model performed better than the second in terms of PP and FN rate, it showed lower BA (0.689 vs. 0.709) and a higher FP rate (16.9% vs. 13.9%) (Figure 5F). The results of the combined model for LGMDR5 mirrored those of the first model (data not shown).

### 3.6 Best Genotype–Phenotype Correlation

The genotype–phenotype correlation model that classified variants affecting the β‐sheets, cadherin‐like, and ATP‐binding domains as AM yielded the highest PP, the second‐highest BA and the most favorable FN/FP rates for LGMDR3. This model resulted in three FN and 11 FP cases. On the other hand, since both β‐ and γ‐sarcoglycans are type II transmembrane proteins that share functional domains and form part of the triple‐helical quaternary structure of the complex, we selected the same model for LGMDR4 and LGMDR5. This model—classifying variants affecting β‐sheets, EGF‐like domains, conserved residues, and protein–protein interactions—produced two FN and 11 FP cases for LGMDR4, and no FN but 13 FP cases for LGMDR5.

Among the FN cases, one patient was homozygous for a missense variant shown not to affect membrane localization in vitro (Table 1). In contrast, 22 of the FP cases involved patients who were homozygous or presumed compound heterozygous for variants demonstrated to impair membrane translocation in functional in vitro assays and/or IHC analyses of homozygous individuals (Table 1). Furthermore, 24 patients with incorrect predictions shared their genotype with other individuals in the cohort who were correctly predicted. Notably, two LGMDR5 FP cases exhibited absent γ‐sarcoglycan on IHC, an unusual finding among clinically mild individuals. Taken together, most incorrect predictions suggest the influence of additional phenotype‐modifying factors beyond the primary disease‐causing variants.

**TABLE 1:** Cases with incorrect phenotype predictions.

Gene	Pa‐ tient ID	Variant 1	Pre‐ dic‐ tion	Evidence of localisation at membrane		Variant 2	Pre‐ dic‐ tion	Evidence of localisation at membrane		Patients' IHC	Correct prediction/s for the genotype^a^
				In vitro assay	IHC of homozygous carriers			In vitro assay	IHC of homozygous carriers		
False negative											
*SGCA*	6	c.92T>C; p. Leu31Pro	AM	Abs.^22^	—	c.583G>A; p.Gly195Arg	LM	—	—	Red. ɑ‐SGC	—
	65	c.229C>T; p. Arg77Cys	AM	Abs.^12^, ^22^, ^25^	Abs.^26^, ^27^, ^28^	c.704C>T; p. Thr235Ile	LM	—	—	—	—
	93	c.371T>C; p. Ile124Thr	AM	Abs.^22^	Abs.^29^, ^30^	c.629 T >C; p.Leu210Pro	LM	—	—	Abs. ɑ‐SGC	—
*SGCB*	148	c.272G>C; p. Arg91Pro	LM	Pres.^22^	Abs.^23^	c.272G>C; p.Arg91Pro	LM	Pres.^22^	Abs.^23^	—	—
	151	c.290G>A; p. Cys97Tyr	LM	—	—	c.656_657del; p.Lys219Serfs*2	AM	—	—	—	—
False positive											
*SGCA*	19	c.229C >T; p. Arg77Cys	AM	Abs.^12^, ^22^, ^25^	Abs.^26^, ^28^	c.157G>A; p. Ala53Thr	AM	—	Abs.^31^/Red.^32^	—	—
	22	c.229C>T; p. Arg77Cys	AM	Abs.^12^, ^22^, ^25^	Abs.^26^, ^28^	c.203G>A; p. Gly68Gln	AM	—	—	—	—
	31	c.229C>T; p. Arg77Cys	AM	Abs.^12^, ^22^, ^25^	Abs.^26^, ^28^	c.229C>T; p. Arg77Cys	AM	Abs.^12^, ^22^, ^25^	Abs.^26^, ^28^	—	26
	32	c.229C>T; p. Arg77Cys	AM	Abs.^12^, ^22^, ^25^	Abs.^26^, ^28^	c.229C>T; p.Arg77Cys	AM	Abs.^12^, ^22^, ^25^	Abs.^26^, ^28^	—	26
	34	c.229C>T; p. Arg77Cys	AM	Abs.^12^, ^22^, ^25^	Abs.^26^, ^28^	c.229C>T; p.Arg77Cys	AM	Abs.^12^, ^22^, ^25^	Abs.^26^, ^28^	—	26
	39	c.229C>T; p. Arg77Cys	AM	Abs.^12^, ^22^, ^25^	Abs.^26^, ^28^	c.229C>T; p. Arg77Cys	AM	Abs.^12^, ^22^, ^25^	Abs.^26^, ^28^	—	26
	44	c.229C>T; p. Arg77Cys	AM	Abs.^12^, ^22^, ^25^	Abs.^26^, ^28^	c.229C>T; p. Arg77Cys	AM	Abs.^12^, ^22^, ^25^	Abs.^26^, ^28^	—	26
	56	c.229C>T; p. Arg77Cys	AM	Abs.^12^, ^22^, ^25^	Abs.^26^, ^28^	c.307A>G; p. Ile103Val	AM	—	—	—	—
	82	c.293G>A; p. Arg98His	AM	Abs.^12^, ^22^	Severely red.^33^	c.293G>A; p. Arg98His	AM	Abs.^12^, ^22^	Severely red.^33^	—	—
	88	c.307A>T; p. Ile103Phe	AM	—	—	c.700G>A; p.Asp234Asn	AM	—	—	—	1
	101	c.488dup; p.Leu164Thrfs*27	AM	—	—	c.661C>T; p. Arg221Cys	AM	—	—	—	—
*SGCB*	136	c.31C>T; p.Gln11*	AM	—	—	c.341C>T; p. Ser114Phe	AM	Abs.^22^	Abs.^34^/Red.^35^	—	2
	137	c.31C>T; p. Gln11*	AM	—	—	c.341C>T; p. Ser114Phe	AM	Abs.^22^	Abs.^34^/Red.^35^	—	2
	140	c.31C>T; p. Gln11*	AM	—	—	c.341C>T; p. Ser114Phe	AM	Abs.^22^	Abs.^34^/Red.^35^	—	2
	146	c.265G>A; p. Val89Met	AM	—	Abs.^36^	c.265G>A; p. Val89Met	AM	—	Abs.^36^	—	—
	147	c.265G>A; p. Val89Met	AM	—	Abs.^36^	c.265G>A; p.Val89Met	AM	—	Abs.^36^	—	—
	154	c.341C>T; p. Ser114Phe	AM	Abs.^22^	Abs.^34^/Red.^35^	c.341C>T; p. Ser114Phe	AM	Abs.^22^	Abs.^34^/Red.^35^	—	10
	159	c.341C>T; p. Ser114Phe	AM	Abs.^22^	Abs.^34^/Red.^35^	c.341C>T; p. Ser114Phe	AM	Abs.^22^	Abs.^34^/Red.^35^	—	10
	168	c.341C>T; p. Ser114Phe	AM	Abs.^22^	Abs.^34^/Red.^35^	c.543C>G; p.Ser181Arg	AM	—	—	—	—
	169	c.341C>T; p.Ser114Phe	AM	Abs.^22^	Abs.^34^/Red.^35^	c.543C>G; p. Ser181Arg	AM	—	—	—	—
	180	c.551A>G; p.Tyr184Cys	AM	—	—	c.551A>G; p.Tyr184Cys	AM	—	—	—	2
	188	c.699_702del; p.Phe233Leufs*16	AM	—	Abs.^35^	c.699_702del; p.Phe233Leufs*16	AM	—	Abs.^35^	—	1
*SGCG*	206	c (385+1_386–1)_ (505+1_506–1)del; p.(del)	AM	—	—	c (385+1_386–1) (505+1_506–1)del; p.(del)	AM	—	—	—	1
	210	c.525del; p.Phe175Leufs*20	AM	—	Abs.^36^, ^37^, ^38^/Pres.^36^	c.525del; p.Phe175Leufs*20	AM	—	Abs.^36^, ^37^, ^38^/Pres.^36^	—	71
	214	c.525del; p.Phe175Leufs*20	AM	—	Abs.^36^, ^37^, ^38^/Pres.^36^	c.525del; p.Phe175Leufs*20	AM	—	Abs.^36^, ^37^, ^38^/Pres.^36^	Abs. γ‐SGC	71
	218	c.525del; p.Phe175Leufs*20	AM	—	Abs.^36^, ^37^, ^38^/Pres.^36^	c.525del; p.Phe175Leufs*20	AM	—	Abs.^36^, ^37^, ^38^/Pres.^36^	—	71
	227	c.525del; p.Phe175Leufs*20	AM	—	Abs.^36^, ^37^, ^38^/Pres.^36^	c.525del; p.Phe175Leufs*20	AM	—	Abs.^36^, ^37^, ^38^/Pres.^36^	—	71
	252	c.525del; p.Phe175Leufs*20	AM	—	Abs.^36^, ^37^, ^38^/Pres.^36^	c.525del; p.Phe175Leufs*20	AM	—	Abs.^36^, ^37^, ^38^/Pres.^36^	—	71
	285	c.525del; p.Phe175Leufs*20	AM	—	Abs.^36^, ^37^, ^38^/Pres.^36^	c (578+1_579‐1) (702+1_703–1)del; p.?	AM	—	Abs.^32^	—	2
	289	c (578+1_579–1) (702+1_703–1) del; p.?	AM	—	Abs.^32^	c (578+1_579‐1) (702+1_703–1)del; p.?	AM	—	Abs.^32^	—	1
	291	c.581 T>C; p.Leu194Ser	AM	Abs.^22^	Abs.^39^/Red.^39^, ^40^	c.581T>C; p. Leu194Ser	AM	Abs.^22^	Abs.^39^/Red.^39^, ^40^	—	2
	293	c.702G>T; p. Met234Ile	AM	—	—	c.702G>T; p. Met234Ile	AM	—	—	Abs. γ‐SGC	—
	295	c.818_821del; p.Tyr273Lysfs*6	AM	—	—	c.818_821del; p.Tyr273Lysfs*6	AM	—	—	—	—
	297	c.848G>A; p.Cys283Tyr	AM	Abs.^22^	Abs.^33^, ^36^, ^41^, ^42^, ^43^	c.848G>A; p.Cys283Tyr	AM	Abs.^22^	Abs.^33^, ^36^, ^41^, ^42^, ^43^	—	19
	307	c.848G>A; p.Cys283Tyr	AM	Abs.^22^	Abs.^33^, ^36^, ^41^, ^42^, ^43^	c.848G>A; p.Cys283Tyr	AM	Abs.^22^	Abs.^33^, ^36^, ^41^, ^42^, ^43^	—	19

Abbreviations: Abs, Absent; AM, absent from the membrane; LM, localized at the membrane; Pres, Present; Red, Reduced.

^a^ Number of correctly predicted patients carrying the same genotype.

Finally, to promote and facilitate the clinical use of these models, we developed an online predictive tool accompanied by a database containing clinical, genotypic and genotype–phenotype correlation data from the present study cohort. This free‐access resource is available at https://cracking‐the‐code.com/. We encourage users to submit their cases and report any discordances between predicted and observed phenotypes to help further refine the models.

## 4 Discussion

In contrast to diseases like Dystrophinopathies—where the genotype–phenotype correlation is primarily based on the presence of truncating vs. non‐truncating variants—disorders predominantly caused by missense variants pose a greater challenge for genotype–phenotype correlation modeling, due to the limited predictability of how these variants impact protein structure and function. This is particularly true for sarcoglycanopathies, where it is challenging to predict the effect of missense variants on protein maturation, complex formation, and membrane trafficking. In this study, we established genotype–phenotype correlation models for LGMDR3, LGMDR4, and LGMDR5, achieving PPs between 80% and 90%, BA from 0.536 to 0.867, and FN rates between 0% and 3.1%.

We firstly confirmed that non‐truncating variants can cause phenotypes ranging from severe to mild. While most patients across all phenotypic groups presented < 30% residual protein on WB, the mild group showed the highest proportion with > 30% remnant protein. Based on previous findings linking > 30% residual protein to later times to loss of ambulation [14], mild cases were predicted to predominantly exhibit such protein levels. Furthermore, since both truncating and non‐truncating variants were linked to severe phenotypes, we would have expected absent protein expression for both variant types. This could be explained by the retention of misfolded sarcoglycans within the ER during quality control mechanisms, a phenomenon not observed with truncated proteins.

Among the tested sarcoglycans, β‐sheet structure was the only shared feature strongly associated with impaired protein trafficking, consistent with a previous mutagenesis study in *SGCB* showing that β‐sheets‐affecting variants are more likely to be pathogenic due to impaired membrane localization [13]. This may relate to the role of β‐sheets in forming and maintaining the tertiary and quaternary structures of the protein and complex, especially the triple‐helical structure formed by β‐, γ‐, and δ‐sarcoglycans. In contrast, α‐helices are largely restricted to transmembrane and intracellular domains—the latter disordered and difficult to model structurally. Predicted benign variants, as shown in the same mutagenesis study, were predominantly found in these regions [13].

Functional domains also significantly influenced the genotype–phenotype correlation model. Cadherin‐like and EGF‐like domains, both involved in cell–cell adhesion through protein–protein interactions, likely contribute to the sarcoglycans' structural role in the DAG complex [20, 21]. This may explain the detrimental effect of variants affecting these domains on membrane localization. Additionally, the EGF‐like domain, like the ecto‐ATPase activity, suggests receptor‐like function and, therefore, their involvement in signaling pathways [6, 7, 21]. This raises the possibility that variants in these domains impair complex function without necessarily preventing membrane localization. Unfortunately, no variants in the ecto‐ATPase domain—strictly related to signaling—were found in homozygous patients with available IHC data, limiting our ability to assess at which stage they impact the protein.

Interestingly, only a few residues were highly conserved according to Evolutionary Trace analysis, and these did not overlap with known functional domains, secondary structures nor intra‐complex protein–protein interaction sites. This observation, coupled with improved performance of models that included conservation for β‐ and γ‐sarcoglycans, suggests that these conserved residues may play important roles in the SC complex's structure or function, perhaps through interactions with other components of the DAG complex, such as dystroglycan and dystrophin.

AlphaFold structural models revealed that intra‐complex protein–protein interactions primarily occur among β‐, γ‐, and δ‐sarcoglycans, with α‐sarcoglycan showing limited interaction with the rest of the complex. This aligns with experimental evidence of the process of complex assembly [5]. Moreover, these interactions occurred between amino acids on or inter‐β‐sheets, reinforcing their role in maintaining the quaternary structure of the complex.

We also caution against over‐reliance on in vitro functional assays for interpreting variant pathogenicity and severity. The comparison between in vitro studies performed in heterologous (HEK293 and HeLa) cell lines, and IHC and WB results from homozygous patients revealed inconsistencies [12, 22]. The most striking contradiction was observed for *SGCA*:c.739G > A; p. Val247Met, which impaired membrane localization in vitro but is usually associated with mild clinical presentations, normal IHC staining, and < 30% protein levels [1, 12, 22]. Conversely, *SGCB*:c.272G > C; p. Arg91Pro showed proper membrane localization in vitro, yet homozygous patients exhibited severe phenotypes, absent β‐sarcoglycan on IHC, and < 30% residual protein [22, 23]. These observations suggest that IHC may be more reliable than in vitro assays and WB for assessing disease severity. This is particularly true for WB, as it does not distinguish between protein located at the membrane and protein retained within the ER. Nevertheless, IHC can be misleading for pathogenicity interpretation of variants associated with mild presentations, since normal IHC results do not indicate that the protein present at the membrane is fully functional.

Accurate prediction of the resulting aberrant transcripts from canonical and non‐canonical splice site variants and their impact on protein expression using *in silico* tools is challenging. As incorrect prediction of the pathogenic mechanism behind these variants could negatively affect model performance, we excluded them from this study. Experimental approaches such as in vitro minigene assays, mRNA analysis, and IHC data would be valuable for clarifying the effects of these variants and enabling their analysis with the best‐fitting models. Additionally, studies comparing *in silico* predictions with experimental results would help assess the accuracy of these tools and improve the predictability of splice site variant effects.

Although the best‐fitting models showed high PP and BA, the presence of FP and FN cases suggests three possible scenarios: (1) inter‐centre variability, including differences in the definition of symptom onset and, consequently, age at onset, (2) the need for further model refinement, or (3) the influence of factors beyond the disease‐causing variant—such as genetic modifiers—on the patient's phenotype [22]. While the marked differences in time to loss of ambulation among patients carrying the same disease‐causing variants point toward the role of genetic modifiers, analyzing a validation cohort would be necessary to distinguish between these scenarios. Unfortunately, this was not currently feasible due to the lack of additional datasets.

To conclude, we established a robust genotype–phenotype correlation framework for Sarcoglycanopathies and developed a free‐access online database and predictive tool to translate genotype information into clinical practice. While further refinements—including the integration of splice‐site variants and interactions with other DAG proteins—are necessary, and validation in independent cohorts and in patients carrying new variants is still required, these models have the potential to enhance clinical prognosis, identify patients at higher risk of rapid progression, personalize care, and allow clinicians to provide reliable expectations about disease progression to the patients and their relatives. Moreover, genetic stratification of patients will enhance clinical trial design and recruitment by minimizing the confounding effects of phenotypic variability and improving outcome reliability—especially in the context of emerging therapies.

## Author Contributions

Conceptualization and data curation: L.L., G.S.K.; formal analysis and visualization: L.L.; resources: G.S.K., J.A.‐P., K.G.C., T.S., G.F.‐E., P.L., N.M., F.D.P., C.A.M.M., E.Z., C.C.W., V.S., J.D.‐M.; software: J.V.‐D.; supervision and funding acquisition: V.S., A.T., J.D.‐M. writing original draft: L.L., A.T., J.D.‐M.; writing review editing: L.L., G.K., J.V.‐D., J.A.‐P., K.G.C., T.S., G.F.‐E., P.L., N.M., F.D.P., C.A.M.M., E.Z., C.C.W., V.S., A.T., J.D.‐M.

## Funding

This work was supported by the Medical Research Council MRC, Project Grant [MR/W019086/1] (to J.D.‐M.) and the Academy of Medical Sciences (AMS) Professorship Scheme [APR4∖1007] (to J.D.‐M). L.L. was supported by the NIHR (NIHR203309) Newcastle Biomedical Research Centre (BRC), a partnership between Newcastle Hospitals NHS Foundation Trust and Newcastle University, funded by the National Institute for Health and Care Research (NIHR). The views expressed are those of the authors and not necessarily those of the NIHR or the Department of Health and Social Care. B.M. was supported by grant NKFIH K 138669.

## Conflicts of Interest

In addition to the funding disclosed in the acknowledgments section, the authors have the following conflicts of interest to disclose. Filipe Di Pace has received honoraria for lectures from Biogen, outside the scope of the current manuscript. Edmar Zanoteli has received honoraria for lectures and presentations from Biogen, Novartis, Sanofi, Roche, and has participated on the Data Safety Monitoring Board or Advisory Board from Biogen, Novartis, Roche. All are outside the scope of the current manuscript. Conrad Weihl has received consulting fees from Sarepta Therapeutics and Mlbio, has participated on the Data Safety Monitoring Board or Advisory Board from Sarepta Therapeutics, and he is editor and chief from Neuromuscular Disorders. All are outside the scope of the current manuscript. Volker Straub has received a research grant from Sarepta Therapeutics and has received consulting fees from Sarepta Therapeutics. All are outside the scope of the current manuscript. Jordi Díaz‐Manera has received consulting fees from Sarepta Therapeutics, Sanofi, Amicus, Spark, Roche, has received honoraria for lectures, presentations, speakers bureaus, manuscript writing or educational events from Sarepta Therapeutics, Sanofi, Amicus, Astellas, has received support for attending meetings and/or travel from Astellas, Sanofi, has patents planned, issued or pending with Boehringer‐Ingelheim, has participated on the Data Safety Monitoring Board or Advisory Board from Sanofi, Sarepta Therapeutics, Amicus, Astellas, Lupin, and has a leadership or fiduciary role in the boards of the World Muscle Society, MYO‐MRI, and The European Pompe Consortium. All are outside the scope of the current manuscript.

The authors declare no conflicts of interest.

## Supporting information


**Figure S1:** Sarcoglycan complex structure modeling.
Output AlphaFold3 models of the sarcoglycan complex using the multimer algorithm with their respective confidence metrics, where α‐sarcoglycan is colored in cyan, β‐sarcoglycan in orange, γ‐sarcoglycan in blue and δ‐sarcoglycan in pink. pTM: predicted template modeling scores, and IpTM: interface predicted template modeling scores.

**Figure S2:** β‐sarcoglycan schematic summary.
Representation of the β‐sarcoglycan protein sequence, with each residue depicted as a rectangle labeled with its one‐letter amino acid code. The figure integrates the following structural and functional information: (i) residues functionally tested in vitro—those impairing membrane translocation (red circles above the amino acid sequence) and those permitting it (green circles above); (ii) secondary structure elements—α‐helices (green) and β‐sheets (yellow); (iii) functional domains (various shades of purple); (iv) structural domains (various shades of green); (v) Evolutionary Trace ranking—highly conserved residues in red and poorly conserved in purple; (vi) residues involved in intra‐complex protein–protein interactions (blue). The position and length of each colored rectangle indicate the specific amino acids involved in each structural or functional feature.

**Figure S3:** γ‐sarcoglycan schematic summary.
Representation of the γ‐sarcoglycan protein sequence, with each residue depicted as a rectangle labeled with its one‐letter amino acid code. The figure integrates the following structural and functional information: (i) residues functionally tested in vitro—those impairing membrane translocation (red circles above the amino acid sequence) and those permitting it (green circles above); (ii) secondary structure elements—α‐helices (green) and β‐sheets (yellow); (iii) functional domains (various shades of purple); (iv) structural domains (various shades of green); (v) Evolutionary Trace ranking—highly conserved residues in red and poorly conserved in purple; (vi) residues involved in intra‐complex protein–protein interactions (blue). The position and length of each colored rectangle indicate the specific amino acids involved in each structural or functional feature.

**Table S1:** Clinical and genetic characteristics of the study cohort.

## Data Availability

The data that support the findings of this study are available from the corresponding author upon reasonable request.

## Sarcoglycan European Cohort Consortium

Adele D′Amico, Adolfo López de Munain, Alicia Alonso‐Jiménez, Ana Camacho‐Salas, Andrea Gangfuß, Andrés Nascimento, Anna Sarkozy, Anneke J. van der Kooi, Arturo Fraga‐Bau, Béla Melegh, Benedikt Schoser, Bjarne Udd, Blaz Koritnik, Carlos Ortez, Chiara Marini Bettolo, Chiara Panicucci, Claudia Weiss, Claudio Bruno, Claudio Semplicini, Cristina Dominguez‐González, Cristina Garrido, David Gómez‐Andrés, Edoardo Malfatti, Elena Pegoraro, Elke De Vos, Francina Munell, Gabriele Dekomien, Giacomo Pietro Comi, Giorgio Tasca, Isabelle Richard, Jan L. De Bleecker, Jana Haberlová, Jesper Helbo Storgaard, Johanna Palmio, John Vissing, Juan Carlos de Leon‐Hernández, Kinga Hadzsiev, Laura Costa‐Comellas, Lea Leonardis, Leroy ten Dam, Lidia González‐Quereda, Luca Bello, Luisa Politano, Manuela Santos, Marianne De Visser, Marie Rohlenová, Matteo Garibaldi, Michela Guglieri, Nicolas Deconinck, Nicoline Løkken, Omar Abdel‐Mannan, Pia Gallano, Roberto Fernández‐Torrón, Ulrike Schara‐Schmidt, Vincenzo Nigro, Vittoria Zangaro.
