# Supplementary figures and images for: Cracking the Code: Genotype–Phenotype Correlation Models in Sarcoglycanopathies
Source: Ann Clin Transl Neurol. 2026 Mar 19:10.1002/acn3.70361. Online ahead of print. doi: 10.1002/acn3.70361 (PMC13395032; doi:10.1002/acn3.70361)

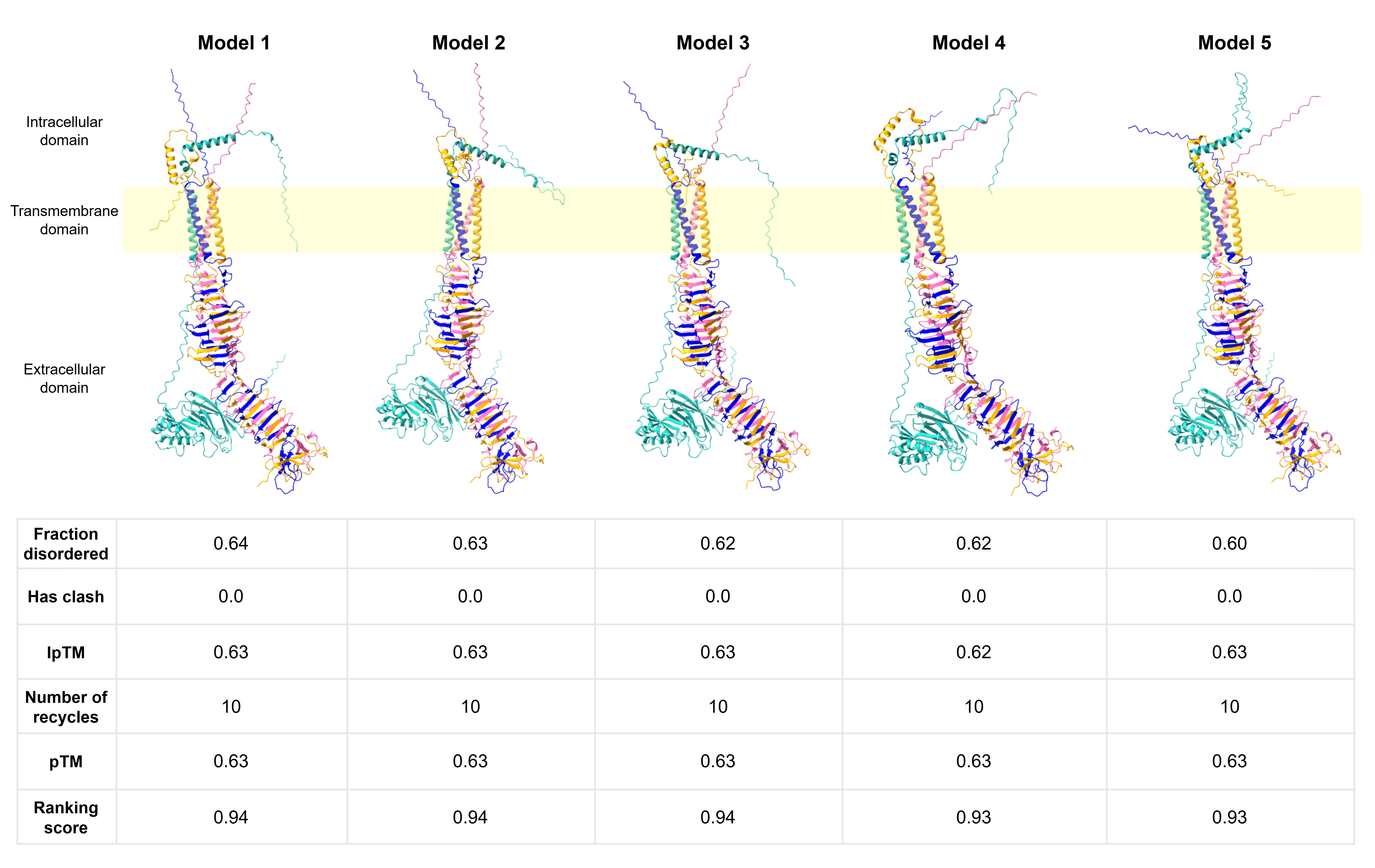

Supplement: Supplementary file 1 — Figure S1: Sarcoglycan complex structure modeling. Output AlphaFold3 models of the sarcoglycan complex using the multimer algorithm with their respective confidence metrics, where α‐sarcoglycan is colored in cyan, β‐sarcoglycan in orange, γ‐sarcoglycan in blue and δ‐sarcoglycan in pink. pTM: predicted template modeling scores, and IpTM: interface predicted template modeling scores. [file ACN3-9999-0-s003.png]

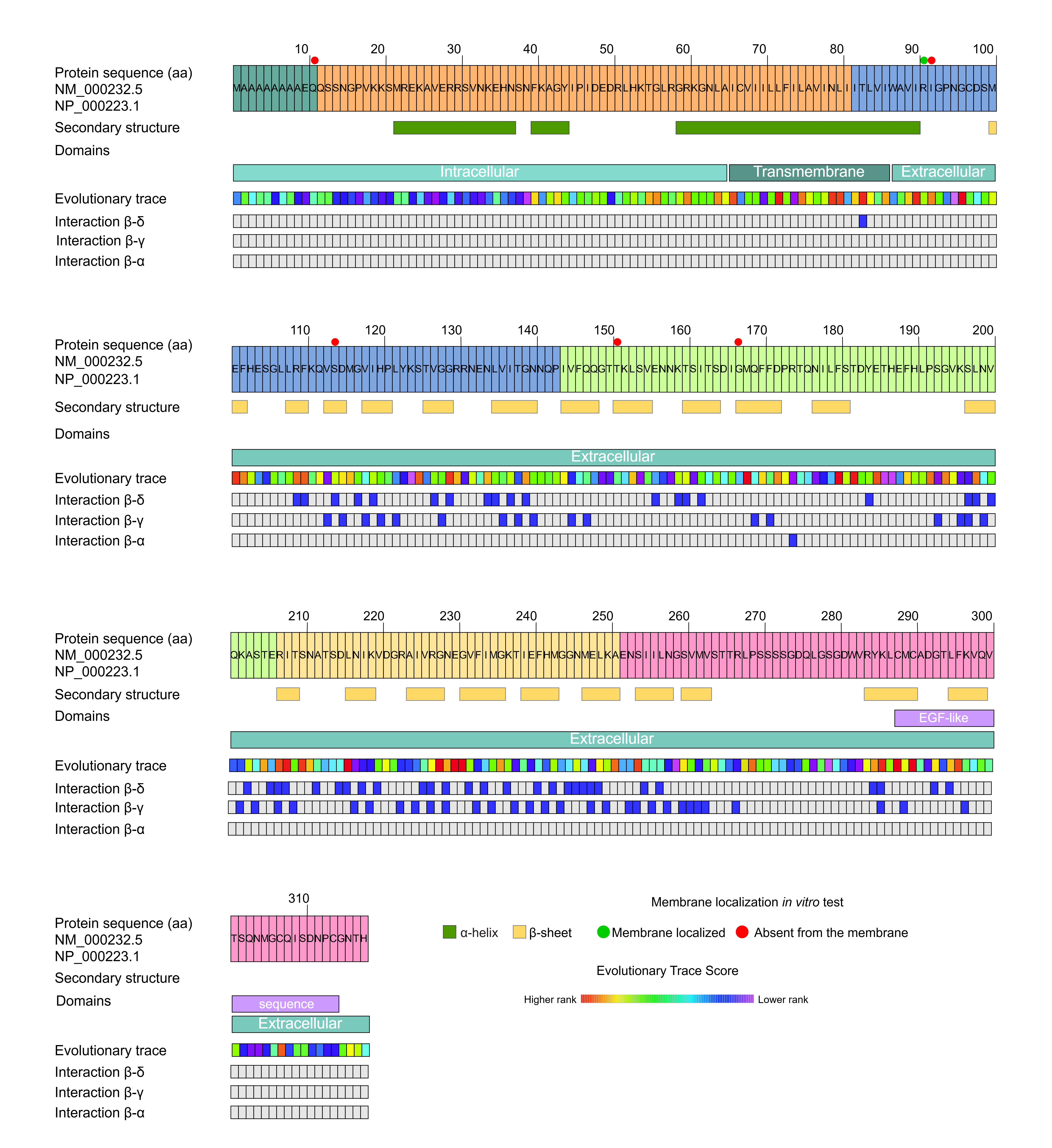

Supplement: Supplementary file 2 — Figure S2: β‐sarcoglycan schematic summary. Representation of the β‐sarcoglycan protein sequence, with each residue depicted as a rectangle labeled with its one‐letter amino acid code. The figure integrates the following structural and functional information: (i) residues functionally tested in vitro—those impairing membrane translocation (red circles above the amino acid sequence) and those permitting it (green circles above); (ii) secondary structure elements—α‐helices (green) and β‐sheets (yellow); (iii) functional domains (various shades of purple); (iv) structural domains (various shades of green); (v) Evolutionary Trace ranking—highly conserved residues in red and poorly conserved in purple; (vi) residues involved in intra‐complex protein–protein interactions (blue). The position and length of each colored rectangle indicate the specific amino acids involved in each structural or functional feature. [file ACN3-9999-0-s002.png]

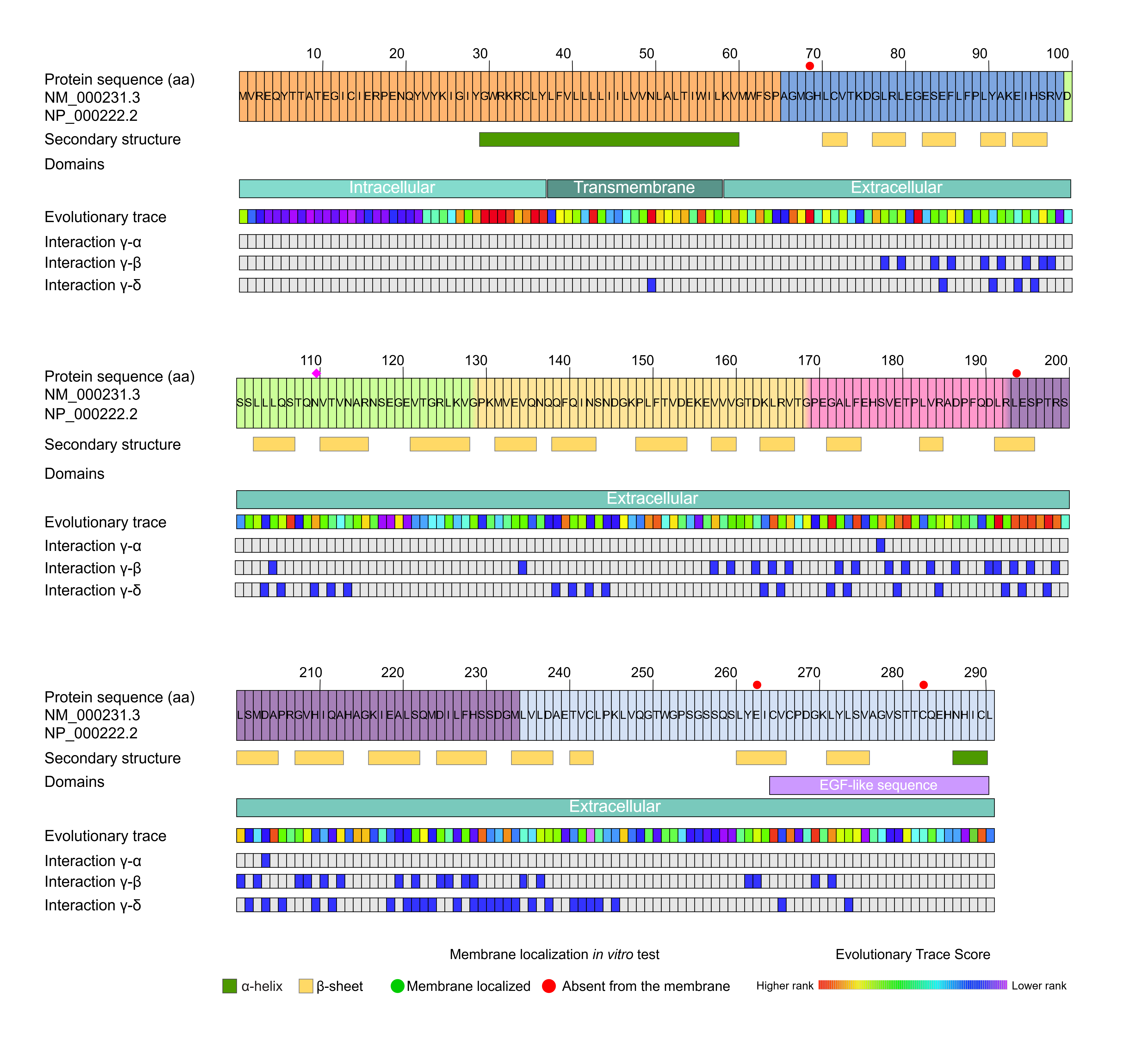

Supplement: Supplementary file 3 — Figure S3: γ‐sarcoglycan schematic summary. Representation of the γ‐sarcoglycan protein sequence, with each residue depicted as a rectangle labeled with its one‐letter amino acid code. The figure integrates the following structural and functional information: (i) residues functionally tested in vitro—those impairing membrane translocation (red circles above the amino acid sequence) and those permitting it (green circles above); (ii) secondary structure elements—α‐helices (green) and β‐sheets (yellow); (iii) functional domains (various shades of purple); (iv) structural domains (various shades of green); (v) Evolutionary Trace ranking—highly conserved residues in red and poorly conserved in purple; (vi) residues involved in intra‐complex protein–protein interactions (blue). The position and length of each colored rectangle indicate the specific amino acids involved in each structural or functional feature. [file ACN3-9999-0-s001.png]
